# Older adults’ experiences of participation in daily activities in Swedish assisted living

**DOI:** 10.1186/s12877-023-04484-y

**Published:** 2023-11-21

**Authors:** Lise-Lotte Jonasson, Angela Bångsbo, Annika Billhult, Maria Wolmesjö

**Affiliations:** 1 Department of Nursing Science, School of Health and Welfare, Jönköping University, Jönköping, Sweden; 2 Faculty of Caring Science, Work Life and Social Welfare, University of Borås, Borås, Sweden

**Keywords:** Assisted living, Interview study, Older adults, Participation, Thematic analysis

## Abstract

**Background:**

According to Swedish law, older adults in Sweden should be able to live a good, safe, and independent life with social and healthcare provided, based on their individual needs. In assisted living in Swedish eldercare the environment affects the older adults’ ability to participate in decision-making and strengthens their ability to feel meaningfulness. The ability of staff working in social and healthcare to invite older adults to participate varies. It is important to examine how older adults perceive their situation, as caregivers in eldercare tend to focus on routine work and experience difficulties in meeting the individual needs of older adults. The aim of this study was to explore how older adults in assisted living experienced participation in daily activities.

**Methods:**

A qualitative interview study was conducted in two municipalities in the western part of Sweden. An exploratory and inductive design was used. Individual interviews were conducted with 11 older adults living in two different assisted living facilities. The data were analysed using thematic analysis.

**Results:**

The interviews resulted in three themes: *Being involved*, *Sense of well-being,* and *Influenced by the context.* The older adults’ experiences of participation were interpreted as feelings of *being involved* in daily life activities, and how they felt involved in their own care and nursing. Participation created prerequisites for well-being. *Sense of well-being* could be related to feelings of security and social community. The organisation and work environment of the healthcare staff had a great impact on their ability to increase the participation of the older adults. The older adults were aware of the everyday work situation of the providers of social and healthcare and were *Influenced by the context.*

**Discussion/conclusion:**

Important conditions for a good quality of life and participation for the older adults are to be treated with respect, receive information, and be able to choose. The older adults expressed several good ideas for improvements and a willingness to be involved in the development of the organisation at different levels. It is essential to invite older adults in assisted living to participate in the design of care and nursing.

## Background

As an older adult, towards the end of life there may be a need for services such as social and healthcare as well as other living arrangements. Various illnesses, impairments and disabilities can gradually make it difficult to manage daily life activities. Social and healthcare become increasingly relevant in this phase of life, as the needs of older adults can be complex due to co-morbidities and frailty [1]. Other factors such as loneliness or cognitive deficits can also affect the complexity of ageing [2].

*Older adults* can be described as people of old age and the definition of an older adult in most developed countries is accepted as an adult who is 65 years of age or older [3]. Older adults represent a heterogeneous group of adults where some have extensive and recurring needs for care and actions from social and healthcare services. The general need for social and healthcare is expected to increase from the age of 80 + [4].

If older adults in Sweden need personal support and qualified social and healthcare there are assisted living set-ups offered, run by the municipalities [5]. Social workers assess the needs of the older adult and possible access to the assisted living facility, based on the Social Service Act [6]. At an assisted living facility, the older adults have their own apartment and access to common areas and care staff members at all hours, factors which may increase the feeling of security. Social care workers, i.e., assessment officers, assistant nurses supported by registered nurses and social work managers provide the social and healthcare. The quality, content, level, and organisation of eldercare can vary between municipalities [5].

The care environment at the assisted living facility affects the older adults’ ability to be involved and decide for themselves, and should strengthen their ability to feel meaningfulness, contribute to reduced suffering, and improve recovery [7]. Hence, healthcare professionals need to identify and adapt to the psychological, social, and existential needs of older adults. This is especially true for older adults who may experience the loss of loved ones, which may influence their identity and autonomy [8]. The needs of older adults living in assisted living vary, from social activities, healthcare, and rehabilitation to end-of-life palliative care, which in turn places great demands on social and healthcare providers [9]. Criteria demonstrating good services and ethical care are that social and healthcare staff should deliver and show older adults respect, invite them to participate, allow self-determination, and provide safe and secure care [10].

The concept of *participation* is complex and can be understood from micro-, meso- and macro perspectives [11]. At the micro level, older adults should be able to participate in meaningful activities with others as they face not only physical and psychological challenges, but also social and existential challenges [12]. This can be troublesome, as shown in a study where 83 percent of surveyed patients felt that their ability to influence healthcare was limited and that the information to be able to participate was inadequate [13]. In a conceptual analysis of the concept of "Patient participation" by Sahlsten [14], the result showed that participation is characterised by an established relationship; that some power or control is handed over by the healthcare staff; shared knowledge and information, as well as active and mutual involvement in intellectual and physical activities. The older adults’ possibilities to participate vary depending on the healthcare staffs’ ability to invite them to participate, and this affects their feeling of self-determination and security [15]. The participation of older adults can be supported and encouraged by the professional healthcare staff creating good care relationships [16]. Previous studies show that being shown respect as well as the healthcare staffs’ ability to invite participation provides a basis for security [15].

At the meso level, participation can be seen from an organisational perspective. At this level, the leader creates the conditions for the nursing staff to carry out the work. In assisted living, first-line managers are responsible for organising and leading the work for healthcare staff. A model to illustrate appropriate leadership for first-line managers with the aim of improving older adults’ participation was developed by Wolmesjö in a former study [17]. According to the model, users can take part at five different levels in the organisation. At a basic level, older adults can be invited to take part in advisory boards, and at the second level, older adults are encouraged to express their opinions to influence decision-making. Moreover, at the third level, older adults can participate in dialogue and planning in operation. At the fourth level, users are seen as “co-producers” and take part in advising boards and meetings on assisted living. The fifth level encompasses equal mandates for older adults and first-line managers [17]. A similar model proposed by Arnstein [18] describes a ladder, where the first step (of a total of five steps) for older adults to participate is to obtain information (step one), followed by consulting (step two), considering possibilities through dialogue (step three), cooperation (step four) and finally, co-determining, which is the last and highest form of participation (step five). When the organisational perspective is prioritised over a patient/user focus, participation is restricted [19]. The concept of participation can also be understood from a macro perspective, i.e., the laws that govern and regulate the social and healthcare of older adults [6, 20].

For eldercare to comply with the Social Services Act [6] and the Health and Medical Service Act [20], it is vital to examine how older adults perceive their situation. This is important to explore, as caregivers in eldercare tend to focus on routine work and experience difficulties in meeting the individual needs of older adults [21]. This, along with older adults’ need for existential conversations to meet existential loneliness, can prove to be difficult to achieve for healthcare staff in assisted living [22]. As the participation and well-being of older adults can be an indicator of the quality of eldercare [23] it is important to explore older adults’ experiences of participation in daily activities. Consequently, as part of a research project called *“Sustainable leadership through participation – Value-based work for increased quality of life in eldercare”* [24], the focus of the present study was to explore how older adults in assisted living experienced participation in daily activities.

## Method

This qualitative interview study was conducted in May and June 2019 in two municipalities in the western part of Sweden. An exploratory and inductive design was used [25]. Individual interviews were conducted by two of the authors (LJ and MW). The data were analysed using thematic analysis [26].

### Participants and selection procedure

To find study participants the authors contacted the first-line managers of two assisted living facilities in a smaller and a medium-sized municipality in the western part of Sweden. Verbal and written information was given about the study, and two first-line managers who, according to Hudson, are called gatekeepers [27] were asked to forward an invitation and information letter about the study to older adults with at least three months experience of living in assisted living. Other inclusion criteria were to be able to verbally conduct an interview and to have no cognitive deficits. The exclusion criteria were cognitive deficits and insufficient knowledge of the Swedish language, and the need for an interpreter during the interview. Eleven older adults, four women and seven men, aged 68–97 years, agreed to participate in the study. The informants all spoke Swedish, were born in Sweden and had lived from a few months up to six years in assisted living. Reasons for moving to assisted living varied among the informants but included feeling insecure in their ordinary living and an increasing need for social- and health services and care due to poor health.

### Data collection

An interview guide was created with inspiration from a questionnaire used in a former study about first-line managers in eldercare [28] and this was further developed in the study project group and discussed with the study reference group. A test interview was conducted and recorded, to assess the logistics, relevance of the questions, and their clarity [25]. The test interview did not lead to any changes. Thus, the test interview was included in the study. See interview guide Table 1.

**Table 1:** Examples of questions from the semi-structured interview guide 2023–10-27

Examples of questions	Examples of probing questions
Who are you? (How will you describe yourself as a person…). Please, would you like to tell us briefly about yourself	Age? Married? Widow/widower? Children? Occupation? When did you move to the assisted living? Need of service/support/care?
Would you please share your experiences of what a typical day might look for you?	Could you please provide further explanation on how a typical day might look for you? Participation in decision making in daily activities, organisation etc.?
Can you describe how you experiences living in an assisted living?	Can you, please explain this further? Can you give an example?
Can you describe and elaborate on your participation in daily activities at the assisted living facility?	What is “participation” for you? Can you provide further explanation about the specifics of the participation in daily activities? Social gathering/community?
Is there anything else you would like to add before ending the interview?	

Before the interview began, the two authors (LJ and MW, who share a professional background in eldercare, and substantial experience of similar interview studies), introduced the older adult with a brief presentation of the study. An open interview technique was used, meaning that the interviews started with an initial question “Would you please tell me about yourself?”. Thereafter, the informants were free to describe and elaborate on their participation in daily activities at the assisted living facility. The introductory questions of an open interview must be directed towards the relevant area and should be simple enough to create a relaxed mood [29]. All interviews ended with the question: “Is there anything else you would like to add before ending the interview?”. The interviews lasted an average time of 38 min (min–max = 10–59 min). One older adult chose to end the interview early (after 10 min) due to fatigue. Data saturation was estimated to have been reached after 10 interviews, as no new information emerged about the phenomenon in interview 11.

### Data analysis

Thematic analysis, a flexible method of analysis that is often used in qualitative studies [26], was used to evaluate the data. The method was used inductively (i.e., data were analysed without a predefined theoretical starting point). Initially, the interviews were transcribed verbatim by LJ. Data were then analysed by two of the authors (LJ and MW), the research group followed the process of analysis, and findings were discussed to ensure data rigour. The analysis consisted of six steps to identify recurring themes from the interviews. Step 1) the text was read several times to gain familiarity with the data corpus; 2) initial coding was performed on the entire dataset, and parts of the data answering the research questions were highlighted; 3) the codes were reread, and themes were generated based on codes relevant to each other; 4) the themes were checked with the extracted codes of each written interview and the corpus data, which generated a thematic map of the analysis; and 5) themes were defined, the specificities of each theme were clarified, and a clear definition and name for the theme was established. Following this, the themes, along with any discrepancies, were discussed with the research group to reach a consensus. These texts were combined into an overall text, and a comprehensive map of all the text was produced. The themes were processed, redefined, and clarified once again to reduce the number of distinct themes. The final step, 6), was to generate the report and present the themes and a coherent pattern.

### Ethical considerations

The study follows the four ethical principles of research: autonomy, not to harm, to do good and the principle of justice [30]. In addition, according to the Helsinki Declaration, the main ethical requirements for research have guided the design of the research study [31]. The older adults were informed about the study and all interviews were conducted with informed consent. The older adults were told their participation was voluntary, that they could end the interview at any time without an explanation, and that they were guaranteed confidentiality and anonymity. The transcribed interviews do not contain any information that can be linked to the older adults as individuals, and the recorded interviews as well as the transcribed text were kept confidential. The study was approved by the Regional Ethical Review Board in Gothenburg, Sweden (Dnr 2019–000801/1109–18).

## Results

The analysis focused on how older adults in assisted living experienced participation in daily activities, and resulted in three themes: *Being involved, Sense of well-being,* and *Influenced by the context.* The themes are presented below with illustrative quotations from the interviews.

### Being involved

The older adults’ experiences of participation were interpreted as feelings of being involved in daily activities. These experiences were described in different ways, varying from the importance of the staff’s approach to the need to have a dialogue with the staff and to obtain information. The older adults were influenced in a good way by the staffs’ behaviour, their competence, their ability to approach the older adults and having an openness for conversations. The informants felt that the healthcare staff had good manners, patience, showed kindness, were willing to serve, and were pleasant and helpful. Moreover, the experience of being shown respect summed up the experience of being involved in daily life activities. Furthermore, the older adults expressed gratitude for receiving service and support with what was needed, although they wished the social and healthcare staff had more time for conversation. Conversation would act as a catalyst for participation, and on one hand, simple acts, such as being informed about the menu and various activities, and being able to influence what, how and when of daily life, would make the older adults feel involved in their care. On the other hand, the older adults felt they did not have to think for themselves and thus received help without being asked. Some of the informants felt that the social and healthcare staff were stressed and, in a hurry, especially in the morning, describing them as “quick as lightning”. However, there was an understanding and acceptance among the older adults that the situation called for that.

They (social and healthcare staff) are really in a hurry in this place; they do not have the time, to be honest (male, 92 years)

I do as much as I can by myself and then I wish they (social and healthcare staff) could sit down and talk with me. They always carry their phone, and when they are with me the phone rings… (female, 92 years).

The older adults in the study reflected upon the concept of participation and said that they felt involved in their own care and in the daily activities at the assisted living facility.

The concept of participation is about the same as co-responsibility. I have responsibility for myself and for what I experience around me. That means I have to say that I think this is very good or I think this is terribly bad (female, 95 years).

The basis for participation was that the older adults received information. In particular, “the food*”* was the most common topic where several of the older adults had been involved in planning the menu. Even "house meetings" to which they were invited by the responsible manager every six weeks, were appreciated by several informants. Participation for the older adults was about getting information before things happened, giving them the opportunity to have a discussion about their own involvement before a decision was made.

### Sense of well-being

The older adults’ experiences of participation created prerequisites for well-being. Well-being could also be related to feelings of security and social community. When living in assisted living, older adults sometimes experience insecurity, unwanted loneliness, and existential issues. The informants in general described sadness about moving to assisted living. Furthermore, the informants even highlighted family, fellowship with others, walking, being able to go outside in nature, good finances and health as important to them.

The well-being of the older adults in the assisted living was described as having the opportunity to participate in common activities and to be part of the surrounding community. Social stimulation and interaction with others were also connected to well-being. Mental stimulation and the opportunity to train the brain were described as important for the older adults. Examples were given, including solving crosswords and work with problem-solving. These factors improved their well-being.

It´s not just about giving old ladies knitting needles for knitting, but really trying to find different and new activities… for example, I would not think life was worth living if I did not have access to books (female, 95).

Well quality of life, I, as a person, like to have people around. I want to talk to people and the staff here is great. I also enjoy the living facilities (male, 97)

We have talked about the need for things to do and they (the social and healthcare staff) have really come through. We have a man, whom I’d believe is retired, that shows up at certain times. He does crossword puzzles with us which is much appreciated for the simple reason that it gives a reason for conversation. I think communication is crucial when living in an assisted living facility (female, 95).

The social and healthcare staff’s ability to invite older adults to participate was important and affected the older adults’ feeling of self-determination and possibilities to participate. Activities like walking and being out in nature were described as important for well-being. Several informants described how it used to be before moving into assisted living and how the outdoor activities had enriched them. Several things such as access to books, different activities, conversing with others and contact with “loved ones”, were described by the older adults as activities that could make them feel healthy and “get people started”. Contact with friends and family by phone or physical visits were described as important links to “being part of society”. Relationships with others gave possibilities for the older adults to share experiences and feel involved.

The informants related the sense of well-being to their thoughts on life and death. On a direct question about whether they could talk to the staff about existential matters, some responded they had special staff to talk to while others had no one to talk to. Furthermore, feelings of anxiety and depression stemming from concerns about one’s own health, and concern for loved ones and the future were mentioned by the informants. The older adults also mentioned difficulties with night sleep when asked questions about the future and the concerns about relatives. The concern was related to a lack of consistency as some healthcare staff treated the concern with a pill and others with a comment that “it pays not to worry”. A certain apathy appeared when informants expressed their thoughts about what to expect in the future. Concerns about losing all bodily and cognitive functions, loneliness and death were brought up. Some informants indicated that fear of loneliness and the wish to be a part of a context were reasons for moving into the assisted living facility. The older adults said that having someone to talk with could minimise the feeling and experience of loneliness, which, in turn, increased the sense of well-being.

No one is under 93, it's a man I think, the vast majority are older. Yes, I hardly talk to them. I don't talk to them, they can't hear… (male, 70 years)

A little concern, and still very much yes. I have maybe only one year left to live maybe…. (male, 92 years)

I choose not to think too much about death until it's time….. I'm pretty sure that if I can still express myself, I'm going to say, 'please do this and that and not this and that', I think so. But I prefer not to give it any thought until it's time (female 95 years).

The understanding of security influenced the older adults’ sense of well-being, and the feelings of safety and security were cited as important reasons for living in the assisted living facility. The knowledge that there were always social- and health care staff nearby mattered, according to the older adults. Being able to be active and to go to the dining room using a walker, and wearing good shoes were mentioned. Another informant said that security was about knowing that a staff member would come when they rang the “alarm bell” for service, support or personal care.

Yesterday, when I was going to the toilet, I had to wait for 20 min before I got assistance and that made me feel insecure; anything could have happened (male, 88 years)

### Influenced by the context

The organisation of the assisted living facility and the work environment of the social and healthcare staff had a great impact on their ability to increase the participation of the older adults. The older adults were aware of the healthcare staff’ everyday work situation and were affected by it. A common description given by the informants was that they felt the social and healthcare staff were in a hurry, often lacking time, which affected the older adults negatively.

Well, they run around and do their chores, and there are large distances to cover. And the internal communication does not work. The security alarm is a joke, it doesn’t feel secure (male, 88).

Consequently, the older adults avoided asking for service, support, and personal care as much as possible. Not being able to go outside for walks or to participate in other activities was mentioned.

Yes, they (social and healthcare staff) do not have time for all their obligations, it is noticeable immediately. Yes, I notice that…. (female, 92 years)

However, in general, the older adults said that the social and healthcare staff were reliable and did what they had promised. The older adults highlighted the importance of good leadership and participation at different levels of the organisation as well as the political level.

She (the manager) does not seem to have the ability to take charge. She should not harass the staff here, she should demand resources from the municipality I guess (male, 88)

The informants said that good leadership was demonstrated by being a good listener and being flexible, thus being able to deal with older adults in different life situations. A certain frustration was expressed about the importance of the assisted living facilities financial resources and that politicians do not listen to the older adults. A call for strong leadership that urged decision-makers to direct more resources to the care of older adults was raised. Meetings with the manager about events in the future were appreciated by the older adults as it was good to get information and have the possibility to discuss upcoming organisational changes before they happened.

Yes, we have a great relationship. X is a great manager who comes by every day and talks to the staff (male, 86 years)

## Discussion

The older adults’ experiences of participation depend on the approach and work situation of the social and healthcare staff as well as support by the managers. There is an understanding by the older adults that they must wait for service, support, and personal care and that not everything will be as they requested. From an individual perspective, there is also an awareness of taking responsibility for what happens in the caring encounter. There is a need and wish to be involved in daily activities and decision-making, both at the individual and group levels. For this to be possible, information from different levels of the social- and health care organisation is required. A sense of well-being can be obtained if time for both daily- and existential conversations with the social and healthcare staff is available. Having the opportunity to socialise with others, to be active, have meaningful activities, and meet loved ones could counteract feelings of loneliness. Furthermore, social and healthcare staff have a responsibility to promote relationships by confirming older adults and making them feel secure.

The social and healthcare staff, as well as first-line managers' work, encompasses creating conditions for a sustainable and health-promoting care environment for older adults, according to the Sustainable Development Goals [32], the Social Service Act [6], and the Health and Medical Service Act [20]. To make this possible, a good work environment according to the Work Environment Act [32] for social and healthcare workers, along with the first line managers is required. The most important factors are collaboration, communication, participation and trust between employees and managers as well as between employers and employees [33]. The need for trust-based leadership has been highlighted at a national level to value the competence and creativity of professionals and increase their ability to make professional decisions [34–36]. Research has shown that it is more difficult to work with people, for instance caring in assisted living, than with objects and especially in combination with lack of recovery during and after work [36]. Several studies have shown that when social and healthcare professionals enjoy a sustainable and positive working environment, addressing the social, environmental, and economic aspects of the organization, older adults perceive the care to be of high quality and report a positive experience of participation [28, 37–39]. This is confirmed by the present study showing that the social and healthcare staff’s work environment relates to how care is conducted and how the older adults experience their sense of participation.

The results of the present study show that there is an acceptance among the older adults to wait for service, support and care. The results revealed that if the daily life activities did not turn out as the older adults wanted, they adapted to the routines. According to Harnett [21], there is a culture that seems limiting regarding influence of the older adults, which can have an impact on how their wishes are accepted and adapted to the prevailing routines. This approach and understanding is also described by Cho et al. [40] who state that health care organisations and policymakers should examine the quality of care within the setting and facilitate the development of strategies to improve quality of life for older adults in assisted living. Strategies could include encouraging meaningful interpersonal relationships, preventing feelings of isolation and limited autonomy, and facilitating acceptance of and adaptation to life in the assisted living facility. The results of the present study show examples of activities presenting opportunities to train the brain and work with problem-solving. Other activities such as walking and being out in nature, access to books, different workshops, conversing with others and contact with “loved ones”, were highlighted as essential. Contact by phone or physical visits were described as giving an important link to the “outside world”. Several suggestions from the older adults such as what could be done to increase the quality of life and “get people started” were raised. Difficulties sleeping were mentioned in the present study. These difficulties can be remedied with a good and meaningful day, leading to a good night’s sleep, as confirmed by Hennawy et al. [41].

The quality of life of older adults is affected by the social stimulation and interaction with others. Surprisingly, in this regard the older adults did not mention conversations with the other older adults in the assisted living facility. It was mainly conversations with social and healthcare staff that were mentioned. The older adults emphasized the importance of social and health care staff having time for conversations. Sundler et al. [42] show that it can sometimes be difficult to make room for deeper communication in a concrete care situation. Therefore, care should be organised so that there is time for fruitful conversations that are quality-enhancing for the older adult, as confirmed by Jonasson et al. [43]. Similarly, the ability of caregivers to connect, and the central aspect of communicating with the older adults should be developed. Responsibility rests on social and healthcare staff and first-line managers and other professionals, using their competence, skill and knowledge regarding different caring tasks, to create confidence for the older adults involved in the caring encounter [44].

In this study, the level of participation and influence among older adults, as discussed in the research by Wolmesjö and Arnstein [17, 18], will be explored. A challenge mentioned by the older adults was that in general, they wanted to take part in group discussions and be active in decisions. The older adults had suggestions on how to enhance their participation and influence in daily life activities. This highlights the importance of the ethical values of first-line managers, and that these values are implemented to support healthcare staff so that in the long run they will benefit the older adults.

According to the informants, they wanted to actively participate, being involved not only in decision-making about their own personal care, but also in decision-making at an organisational level. The older adults were positive about attending and participating at organisational level and had several ideas on what needed to be changed or further developed in the organisation. Surprisingly, they were aware of the daily challenges and working conditions of the healthcare staff and the organisational structure. From the older adults’ perspective, participation was mainly discussed in relation to the social and healthcare staff and the older adult as an individual. Our study points out that older adults want to stay active and participate and be part of society as long as possible. This may be important to consider in policies aimed at motivating older adults towards collective co-production [45]. From social, health and activity perspectives this needs to be prioritised and leaders in the care of older adults must be given resources to lead, which would result in higher user participation. Perhaps the model of Wolmesjö & Kullén Engström [17] could be successful and increase the older adults’ possibilities to participate in encounters with staff, other older adults, and wider society. Overall, this study shows that older adults' participation in daily activities in assisted living takes place at the personal (micro) level, group level (meso) and at the societal (macro) level [11].

Compared to a national level, men were overrepresented in this group. The results may have been different if both genders were equally represented, as gender differences regarding health and well-being in late life exist [46]. Furthermore, older adults as a group often have various diseases and thus could be seen as persons in a vulnerable situation. However, the older adults in this study were required to have the mental capacity to make their own decision about whether to participate in the interview situation or not; they could end the interview at any time without an explanation in accordance with Beauchamp and Childress [30]. One older adult chose to end the interview after 10 min due to fatigue. It is always the researcher’s duty to act ethically and flexibly; therefore, this interview was included in the analysis. By using a gatekeeper (i.e. a first-line manager) to identify possible older adults to invite into the study, we were supported by the organisational level to carry out this study. When the persons were selected, a personal invitation was sent to the informants by the researchers (LJ and MW), including written information about the project and the study. The gatekeeper’s role then was to facilitate a physical meeting. However, Hudson [27] has found that the gatekeeper can obstruct data collection. In this study, the gatekeeper provided invaluable help, without interfering with or obstructing the research process. To promote thoroughness in this study, a systematic approach to the research method as proposed by Braun & Clarke [26] has been followed. Bearing in mind the well-being of the informants, it was important for the researchers to avoid interview situations that created aroused emotions. Data saturation was estimated to have been reached after 10 interviews, as no new information emerged about the phenomenon in interview 11. However, it is always possible that new information would emerge, should several more interviews have been carried out. The data obtained after 11 interviews was deemed to be sufficient for analysis.

## Conclusion

The results show that important conditions facilitating the participation of older adults residing in assisted living are to be treated with respect, receive information, and be able to make individual choices. Other important conditions are to experience security as well as care provided in a safe environment. The older adults expressed several ideas for improvements and showed a willingness to be involved in the development of the organisation at different levels. At the same time, there was a great awareness of what the work situation was like for the social and healthcare staff, and therefore the participants tried "not to disturb" or make demands. Requirements for older adults to be able to participate in daily activities could be considered reasonable,or example, to be able to influence and follow the daily and annual rhythm and choice of activities based on individual health and the weather. The study highlights the benefits of inviting older adults in assisted living to participate in the design of care and nursing and could be used by decision-makers in the municipality in future roadmaps to improve healthcare.

## Data Availability

The dataset from this study is available from the corresponding author upon reasonable request.
